# An Experimental Group A *Streptococcus* Vaccine That Reduces Pharyngitis and Tonsillitis in a Nonhuman Primate Model

**DOI:** 10.1128/mBio.00693-19

**Published:** 2019-04-30

**Authors:** Tania Rivera-Hernandez, Diane G. Carnathan, Scott Jones, Amanda J. Cork, Mark R. Davies, Peter M. Moyle, Istvan Toth, Michael R. Batzloff, James McCarthy, Victor Nizet, David Goldblatt, Guido Silvestri, Mark J. Walker

**Affiliations:** a Australian Infectious Diseases Research Centre, The University of Queensland, St Lucia, QLD, Australia; b School of Chemistry and Molecular Biosciences, The University of Queensland, St Lucia, QLD, Australia; c Emory Vaccine Center, Emory University, Atlanta, Georgia, USA; d Yerkes National Primate Research Center, Emory University, Atlanta, Georgia, USA; e Great Ormond Street Institute of Child Health, University College London, London, United Kingdom; f Peter Doherty Institute, University of Melbourne, Parkville, VIC, Australia; g School of Pharmacy, The University of Queensland, St Lucia, QLD, Australia; h Institute for Glycomics, Griffith University, Gold Coast, QLD, Australia; i Australian Infectious Diseases Research Centre, QIMR Berghofer Medical Research Institute, Brisbane, QLD, Australia; j Division of Host-Microbe Systems and Therapeutics, Department of Pediatrics, University of California—San Diego, La Jolla, California, USA; Max Planck Institute for Infection Biology; University of Auckland; Helmholtz Centre for Infection Research (HZI)

**Keywords:** *Streptococcus pyogenes*, group A *Streptococcus*, immunization, nonhuman primates

## Abstract

Group A *Streptococcus* (GAS) infections account for an estimated 500,000 deaths every year. This bacterial pathogen is responsible for a variety of mild and life-threatening infections and the triggering of chronic autoimmune sequelae. Pharyngitis caused by group A *Streptococcus* (GAS), but not asymptomatic GAS carriage, is a prerequisite for acute rheumatic fever (ARF). Repeated bouts of ARF may trigger rheumatic heart disease (RHD), a major cause of heart failure and stroke accounting for 275,000 deaths annually. A vaccine that prevents pharyngitis would markedly reduce morbidity and mortality from ARF and RHD. Nonhuman primates (NHPs) have been utilized to model GAS diseases, and experimentally infected rhesus macaques develop pharyngitis. Here we use an NHP model of GAS pharyngitis to evaluate the efficacy of an experimental vaccine, Combo5 (arginine deiminase [ADI], C5a peptidase [SCPA], streptolysin O [SLO], interleukin-8 [IL-8] protease [SpyCEP], and trigger factor [TF]), specifically designed to exclude GAS components potentially linked to autoimmune complications. Antibody responses against all Combo5 antigens were detected in NHP serum, and immunized NHPs showed a reduction in pharyngitis and tonsillitis compared to controls. Our work establishes the NHP model as a gold standard for the assessment of GAS vaccines.

## Abstract (precis)

GAS-related diseases disproportionally affect disadvantaged populations (e.g., indigenous populations), and development of a vaccine has been neglected. A recent strong advocacy campaign driven by the World Health Organization and the International Vaccine Institute has highlighted the urgent need for a GAS vaccine. One significant obstacle in GAS vaccine development is the lack of a widely used animal model to assess vaccine efficacy. Researchers in the field use a wide range of murine models of infection and *in vitro* assays, sometimes yielding conflicting results. Here we present the nonhuman primate pharyngeal infection model as a tool to assess vaccine-induced protection against colonization and clinical symptoms of pharyngitis and tonsillitis. We have tested the efficacy of an experimental vaccine candidate with promising results. We believe that the utilization of this valuable tool by the GAS vaccine research community could significantly accelerate the realization of a safe and effective GAS vaccine for humans.

## INTRODUCTION

Acute rheumatic fever (ARF) leading to rheumatic heart disease (RHD) causes significant global morbidity and mortality, with an estimated prevalence of 32 million cases, resulting in 275,000 deaths each year (1, 2). With over 2.4 million cases in children, RHD is the most common pediatric heart affliction (3, 4). It is well established on clinical and epidemiological grounds that group A *Streptococcus* (GAS) (Streptococcus pyogenes) pharyngitis is a precursor for ARF (5). A vaccine to address the global burden of ARF and RHD must therefore reduce GAS pharyngitis (6); however, a safe and effective commercial vaccine for this purpose is lacking. Obstacles hindering GAS vaccine development include strain diversity, antigenic variation, differences in the geographical distribution of serotypes, and the potential for GAS antigens to trigger autoimmune sequelae, including rheumatic fever (7). In 1979, the U.S. Food and Drug Administration placed a ban on GAS vaccine development research using human subjects that lasted two and a half decades. The 2004 workshop instrumental in lifting this moratorium implicated structural features of the surface-anchored M protein and the *N*-acetylglucosamine (GlcNAc) side chain of the species-defining cell wall group A carbohydrate (GAC) in molecular mimicry of host components, potentially provoking autoimmunity and development of ARF (8). As one consequence of the earlier FDA moratorium, only a limited number of GAS vaccine candidates have progressed to phase I and II clinical trials, and to date, evidence for potential vaccine-induced protection stems only from preclinical studies using rodents and *in vitro* assays (9–11).

Pharyngitis, the most common GAS disease manifestation in humans, cannot be reproduced in mice. However, spontaneous GAS pharyngeal carriage in rhesus macaques has been documented (12). Nonhuman primates (NHPs) experimentally infected with GAS in the upper respiratory tract develop pharyngitis and tonsillitis clinical signs such as erythema, palatal petechiae, and occlusion of the oropharyngeal space (13, 14). Here we report the development of a pharyngeal infection model in rhesus macaques to assess vaccine efficacy against GAS pharyngitis and evaluation of the protective efficacy a non-M-protein-based vaccine candidate (Combo5) in the NHP model.

## RESULTS AND DISCUSSION

This work presents the NHP pharyngeal infection model as a valuable tool to advance GAS vaccine research. Pharyngitis, the most common GAS disease manifestation, which is prerequisite for the development of ARF, cannot be modeled in mice. Pharyngitis is also relevant as it represents a potential clinical endpoint in a clinical trial setting for GAS vaccine candidates. In order to optimize GAS pharyngeal infection in NHPs, we first performed a small pilot experiment to determine the GAS infecting dose that yielded colonization of the upper respiratory tract and development of pharyngitis and tonsillitis clinical signs. We used the representative GAS M1T1 5448 strain; the GAS M1T1 clone is prevalent and globally disseminated, responsible for both mild and severe infections (15). Under anesthesia, two NHPs were intranasally infected with either 1 × 10^7^ or 5 × 10^7^ CFU of GAS M1T1 5448. NHPs were scored by veterinary staff for clinical signs using an established pharyngitis and tonsillitis scoring system (see Table S1 in the supplemental material) on days 1, 2, 3, 7, 14, 21, and 28 following infection. Using a dose of 5 × 10^7^ CFU, reproducible culture-positive GAS was established, and clinical signs of disease were observed (Table S2). This dose was used in subsequent experiments.

10.1128/mBio.00693-19.5 TABLE S1 Scoring system for pharyngitis and tonsillitis symptoms (J. M. Skinner, I. C. Caro-Aguilar, A. M. Payne, L. Indrawati, et al., Microb Pathog 50:39–47, 2011, https://doi.org/10.1016/j.micpath.2010.10.004). Download Table S1, PDF file, 0.03 MB. Copyright © 2019 Rivera-Hernandez et al. 2019 Rivera-Hernandez et al. This content is distributed under the terms of the Creative Commons Attribution 4.0 International license.

10.1128/mBio.00693-19.6 TABLE S2 Colonization, pharyngitis, and tonsillitis symptoms in pilot experiments. Download Table S2, PDF file, 0.03 MB. Copyright © 2019 Rivera-Hernandez et al. 2019 Rivera-Hernandez et al. This content is distributed under the terms of the Creative Commons Attribution 4.0 International license.

**FIG 1:**
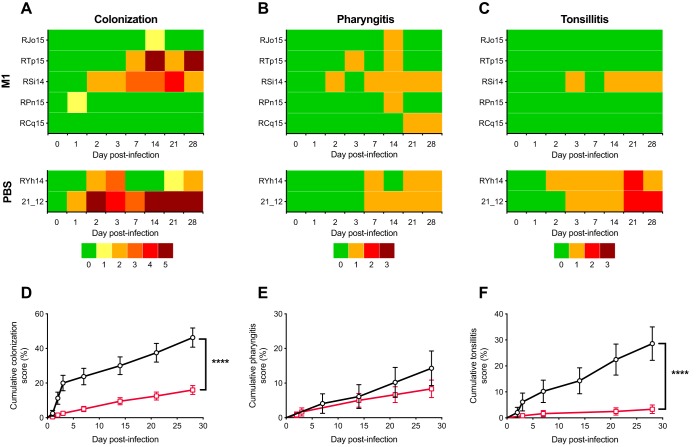
M1 immunization protects against GAS colonization in an NHP model for pharyngeal infection. NHPs were immunized on weeks 0, 8, and 17 with M1 (*n* = 5) and PBS (*n* = 2). On week 20, NHPs were intranasally infected with 5 × 10^7^ CFU of GAS M1T1 5448. Individual NHP identifiers are indicated on the left. (A to C) Individual scores for colonization (A), pharyngitis (B), and tonsillitis (C) were recorded on days 1, 2, 3, 7, 14, 21, and 28 postinfection. (D to F) Log rank analysis of grouped cumulative scores for colonization (D), pharyngitis (E), and tonsillitis (F) following GAS infection was performed to compare protection afforded by M1 immunization (red lines) and that in PBS-immunized control NHPs (black lines). Values represent cumulative score percentages ± standard errors (SE) (****, *P < *0.0001).

It is well established that as a consequence of natural infection, an immune response against the preeminent GAS virulence factor, M protein, provides serotype-specific protection against the same GAS M serotype (16). Serotype-specific protection was also observed in a series of experimental human infections carried out in the 1970s, where immunization with purified native M1 protein protected human volunteers against symptoms of illness, including fever, erythema, and exudative pharyngitis (17). Homologous M protein vaccination and challenge are also commonly used as a positive control in murine vaccination models (18–20). To validate the NHP model as a tool for GAS vaccine development, we tested whether serotype-specific protection could be observed. Rhesus macaques (*n* = 5) were immunized with purified M1 protein adjuvanted with alum on weeks 0, 8, and 17. Compared to phosphate-buffered saline (PBS)-alum negative controls (*n* = 2), specific anti-M1 antibodies were detected in serum samples from M1-vaccinated NHPs following primary immunization, and antibody titers increased further after booster immunizations (Fig. S1A). At week 20, NHPs were intranasally infected with 5.2 × 10^7^ CFU of M1T1 GAS strain 5448 and assessed up to 28 days postinfection. Body temperature, weight, and white blood cell (WBC) counts were monitored during GAS pharyngeal infection, which was well tolerated, with no significant clinical indication of an adverse systemic reaction to GAS infection observed among groups (Fig. S1B to D). Throat swabs taken at various time points during infection were assigned a colonization score (Table S3) for each individual NHP (Fig. 1A), in addition to veterinary scoring for pharyngitis and tonsillitis clinical signs (Fig. 1B and C). Cumulative scores for colonization and tonsillitis were significantly lower for NHPs immunized with purified M1 protein than for PBS-immunized controls (Fig. 1D and F). While pharyngitis scores were lower for M1-immunized NHPs than for PBS-immunized macaques, these did not achieve statistical significance (Fig. 1E). Postinfection serum samples from PBS-immunized NHPs displayed increased anti-M1 antibody titers (Fig. S1A, shaded area), suggesting the development of serotype-specific immunity following GAS infection of naive NHPs. Taken together, these observations suggest that the NHP infection model can reproduce vaccine-induced serotype-specific immunity, paralleling observations reported in humans and providing a platform to assess the efficacy of experimental GAS vaccines to prevent pharyngitis.

10.1128/mBio.00693-19.1 FIG S1 Antibody response and animal welfare measures following infection of M1-immunized NHPs. (A) Serum samples from M1-immunized (*n* = 5) (red lines) and PBS-immunized (*n* = 2) (black lines) NHPs were collected before each immunization on weeks 0, 8, and 17 and postinfection on week 20 (days 0, 1, 2, and 3 postinfection), week 21 (day 7 postinfection), week 22 (day 14 postinfection), week 23 (day 21 postinfection), week 24 (day 28 postinfection), and week 25 (1 week after antibiotic treatment). Antibody titers at the time of infection (week 20) were significantly higher in M1-immunized NHPs than in PBS-immunized NHPs (*P* < 0.0001). Infection and antibiotic treatment days are marked by red and green arrows, respectively. Duration of GAS infection is indicated by the gray-shaded area. Values represent the geometric mean titers ± geometric SD. (B to D) Welfare of NHPs during the course of infection was monitored by measuring rectal temperature (B), weight (C), and white blood cell counts (D). Symbols represent the mean values ± SD. Download FIG S1, PDF file, 0.1 MB. Copyright © 2019 Rivera-Hernandez et al. 2019 Rivera-Hernandez et al. This content is distributed under the terms of the Creative Commons Attribution 4.0 International license.

10.1128/mBio.00693-19.7 TABLE S3 Colonization scoring system (E. M. Dunne, J. L. Marshall, C. A. Baker, J. Manning, et al., BMC Infect Dis 13:312, 2013, https://doi.org/10.1186/1471-2334-13-312). Download Table S3, PDF file, 0.03 MB. Copyright © 2019 Rivera-Hernandez et al. 2019 Rivera-Hernandez et al. This content is distributed under the terms of the Creative Commons Attribution 4.0 International license.

**FIG 2:**
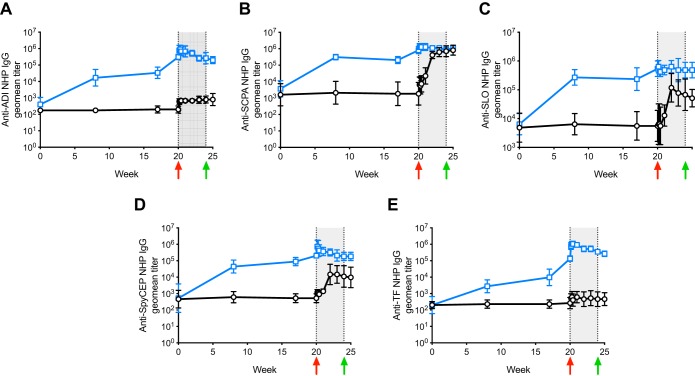
Combo5 antigen-specific IgG response in NHP serum. Serum samples from Combo5-immunized (*n* = 5) (blue lines) and PBS-immunized (*n* = 5) (black lines) NHPs were collected before each immunization on weeks 0, 8, and 17; prior to infection on week 20; and postinfection on week 20 (days 1, 2, and 3 postinfection), week 21 (day 7 postinfection), week 22 (day 14 postinfection), week 23 (day 21 postinfection), week 24 (day 28 postinfection), and week 25 (1 week after antibiotic treatment). Antibody titers at the time of infection (week 20) were significantly higher for all antigens in Combo5-immunized NHPs than in PBS-immunized NHPs (*P < *0.0001). Infection and antibiotic treatment days are marked by red and green arrows, respectively. Duration of GAS infection is indicated by the gray-shaded area. IgG responses against ADI (A), SCPA (B), SLO (C), SpyCEP (D), and TF (E) were induced in Combo5-immunized NHPs following immunization. Infection with GAS M1T1 5448 significantly raised IgG titers against SCPA (B), SLO (C), and SpyCEP (D) antigens in PBS-immunized NHPs. Values represent the geometric mean (geomean) titers ± geometric SD.

**FIG 3:**
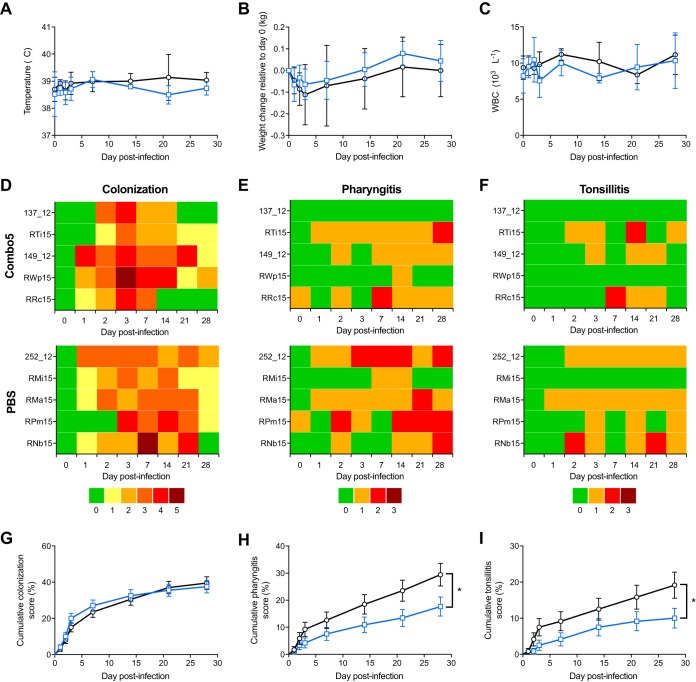
Combo5 immunization reduces pharyngitis and tonsillitis. (A to C) Welfare of NHPs during the course of infection was monitored by measuring rectal temperature (A), weight (B), and white blood cell counts (C) for PBS-immunized (*n* = 5) (black lines) and Combo5-immunized (*n* = 5) (blue lines) NHPs. Symbols represent the mean values ±SD. (D to F) Individual scores for colonization (D), pharyngitis (E), and tonsillitis (F) following infection of Combo5- and PBS-immunized NHPs with 5 × 10^7^ CFU of GAS M1T1 5448. Individual NHP identifiers are indicated on the left. (G to I) Log rank analysis of grouped cumulative scores for colonization (G), pharyngitis (H), and tonsillitis (I) following GAS infection was performed to compare protection afforded by Combo5 immunization (blue lines) to that in PBS-immunized control NHPs (black lines). Values represent cumulative score percentages ± SE (*, *P < *0.05).

We next used the NHP infection model to assess the immunogenicity and efficacy of a non-M protein GAS vaccine. Vaccine candidate Combo5 (18) is composed of five GAS protein antigens (arginine deiminase [ADI] [20, 21], trigger factor [TF] [20], C5a peptidase [SCPA] [22], interleukin-8 [IL-8] protease [SpyCEP] [19], and streptolysin O [SLO] [19, 23]) with proven protective efficacy in mouse models. This formulation excludes GAS components M protein and the GlcNAc antigen of group A carbohydrate that are potentially linked to autoimmune complications in humans (8, 24). NHPs (*n* = 5 per group) were immunized with 100 μg of Combo5 (20 μg each antigen) or PBS formulated in alum. Antibody titers against all five antigens increased following primary immunization (week 0) and subsequent booster immunizations (weeks 8 and 17) in Combo5-immunized NHPs (Fig. 2), while antigen-specific antibody titers remained unchanged in PBS-alum-immunized NHPs throughout the immunization regime. At week 20, NHPs were intranasally infected with ∼5 × 10^7^ CFU of GAS strain 5448 (range, 4.95 × 10^7^ to 5.22 × 10^7^ CFU). Similar to the immune response to human GAS infection (25–27), PBS-alum-immunized NHPs mounted a gradual increase in anti-SCPA, anti-SLO, and anti-SpyCEP antibody titers following GAS infection (Fig. 2B to D, shaded area). On the other hand, antibodies against ADI and TF, which are not detected in sera from convalescent human infection (20), were not elevated in control PBS-alum-immunized NHPs postinfection (Fig. 2A and E, shaded area). The levels of a select panel of inflammation markers (IL-6, interferon gamma-induced protein 10 [IP-10], IL-1β, IL-17A, interferon beta [IFN-β], IL-23, tumor necrosis factor alpha [TNF-α], IFN-γ, granulocyte-macrophage colony-stimulating factor [GM-CSF], IL-8, and monocyte chemoattractant protein 1 [MCP-1]) measured in NHP serum postchallenge were comparable among all NHP groups (Fig. S2). Following GAS challenge, body temperature (Fig. 3A), changes in body weight (Fig. 3B), and white blood cell counts (Fig. 3C) did not differ between Combo5-alum and PBS-alum groups. Individual scores for colonization (Fig. 3D), pharyngitis (Fig. 3E), and tonsillitis (Fig. 3F) were recorded across the course of infection. All NHPs in this study developed culture-positive GAS infection by day 3 postinfection. Five of five PBS control NHPs and 4/5 Combo5-vaccinated NHPs developed pharyngitis, with 4/5 PBS control and 3/5 Combo5-vaccinated NHPs also developing tonsillitis. While no difference was observed in cumulative scores for colonization between Combo5-alum- and PBS-alum-immunized NHPs (Fig. 3G), Combo5 immunization significantly reduced cumulative scores for pharyngitis (Fig. 3H) and tonsillitis (Fig. 3I). All NHPs developed a significant increase in anti-M1 antibody titers in response to infection (Fig. S3). The increase in antibody titers was comparable between both groups.

10.1128/mBio.00693-19.2 FIG S2 Inflammation markers during GAS infection. Serum samples from Combo5 (blue lines)-, M1 (red lines)-, and PBS (black lines)-immunized NHPs collected during GAS infection were used to detect inflammation markers. A multiplex assay was used to detect IL-6 (A), IP-10 (B), IL-1β (C), IL-17A (D), IFN-β (E), IL-23 (F), TNF-α (G), IFN-γ (H), GM-CSF (I), IL-8 (J), and MCP-1 (K). Samples were analyzed in duplicate, and symbols represent the mean values ± SD. Download FIG S2, PDF file, 0.1 MB. Copyright © 2019 Rivera-Hernandez et al. 2019 Rivera-Hernandez et al. This content is distributed under the terms of the Creative Commons Attribution 4.0 International license.

10.1128/mBio.00693-19.3 FIG S3 Development of anti-M1 antibodies in response to infection. (A) Serum samples from PBS-immunized (*n* = 5) (black lines) and Combo5-immunized (*n* = 5) (blue lines) NHPs were collected at days 0, 1, 2, 3, 7, 14, 21, 28, and 35 postinfection. The green arrow indicates antibiotic treatment. Values represent the geometric mean titers ± geometric SD. (B) Anti-M1 antibody titers at the end of the experiment (day 35 postinfection) were significantly higher than those prior to infection (day 0) for all PBS- and Combo5-immunized NHPs. Download FIG S3, PDF file, 0.1 MB. Copyright © 2019 Rivera-Hernandez et al. 2019 Rivera-Hernandez et al. This content is distributed under the terms of the Creative Commons Attribution 4.0 International license.

**FIG 4:**
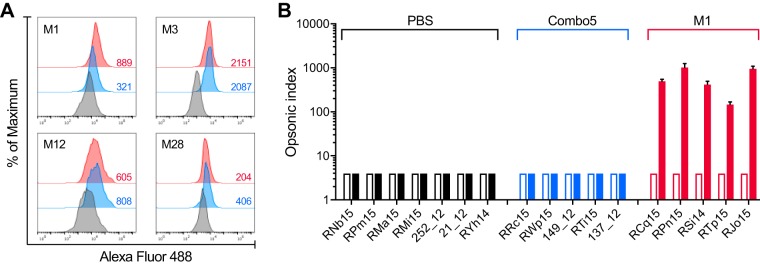
Antibodies against Combo5 antigens bind to the surface of live GAS but do not enhance killing by HL-60 cells. (A) Pooled serum from PBS (black lines)-, Combo5 (blue lines)-, and M1 (red lines)-immunized NHPs was incubated with live M1, M3, M12, and M28 GAS strains. Binding was detected by flow cytometry, and *T*(*X*) values, shown on the lower right side of each histogram, were determined by the probability binning algorithm in FlowJo comparing samples incubated with Combo5 and M1 immune sera versus PBS immune sera. A cutoff value for the statistic *T*(*X*) was established by comparing *T*(*X*) values between sample replicates, and a *T*(*X*) value of >200 was considered significant (*P < *0.01). (B) Antibody functionality of PBS (black bars), Combo5 (blue bars), and M1 (red bars) immune NHP sera was tested using a standardized *in vitro* opsonophagocytic assay. Differentiated HL-60 cells were incubated in the presence of GAS M1 strain 43, a source of complement, and NHP sera from preimmunization (clear bars) and postimmunization (solid bars) time points. The opsonic index was calculated as the serum dilution where 50% killing of bacteria occurred. Immunization with M1 increased significantly (*P < *0.0001) the ability of serum antibodies to enhance killing of GAS, while Combo5 did not. Bars represent the opsonic index ± SD.

The capacity of elicited NHP serum antibodies to bind to the surface of live GAS was assessed using flow cytometry. Four different GAS serotypes (M1, M3, M12, and M28) were chosen for this assay, as they are epidemiologically relevant (28) and belong to different *emm* clusters (29). Immune sera from Combo5- and full-length M1 protein-immunized NHPs produced significant shifts in fluorescence compared to PBS control sera for all serotypes examined (Fig. 4A), indicating recognition and binding of antigen-specific antibodies to the GAS surface. Immune sera from full-length M1 protein-immunized NHPs promoted opsonophagocytic killing (OPK) of serotype M1 GAS (>70%), whereas Combo5-alum and PBS-alum sera did not (Fig. 4B). These data suggest that while Combo5-alum promotes an antibody response that recognizes the GAS cell surface, the reduction in pharyngitis and tonsillitis afforded by Combo5-alum vaccination does not depend *per se* on high levels of opsonic antibody. On the other hand, vaccination with full-length M1 protein results in opsonic antibody and prevents colonization by homologous serotype M1 GAS. Opsonic antibodies against M protein are thought to be a potential correlate of protection in serotype-specific immunity (16); however, it is unclear if this paradigm applies to non-M protein antigens. Effector function assays with Combo5-alum and PBS-alum sera showed that antibodies present in Combo5-alum sera were able to inhibit red blood cell (RBC) hemolysis by SLO (Fig. S4A) and IL-8 degradation by SpyCEP (Fig. S4B), virulence mechanisms which contribute to GAS pathogenesis. While an ideal GAS vaccine would reduce colonization, Combo5 is the first non-M protein vaccine that has shown efficacy in reducing pharyngitis. Further studies are required to determine the role of opsonic antibodies in vaccine-induced protection against GAS pharyngitis and colonization.

10.1128/mBio.00693-19.4 FIG S4 Effector functions of Combo5 antibodies. (A) Pooled serum from Combo5 (blue lines)-immunized NHPs was able to inhibit SLO hemolytic activity compared to serum from PBS (black lines)-immunized NHPs. Hemolytic activity of SLO in the absence of serum was used as 100%. Values represent the means ± SD. (B) Serum from Combo5-immunized NHPs (blue bars) significantly inhibited SpyCEP-mediated IL-8 degradation compared to serum from PBS-immunized NHPs (white bars) after 2 and 4 h of coincubation with live GAS. Bars represent the means ± SD. Residual IL-8 concentrations were compared using two-way ANOVA with Sidak’s multiple-comparison test (**, *P* < 0.005; ***, *P* < 0.001). Download FIG S4, PDF file, 0.1 MB. Copyright © 2019 Rivera-Hernandez et al. 2019 Rivera-Hernandez et al. This content is distributed under the terms of the Creative Commons Attribution 4.0 International license.

During the 71st World Health Assembly, the World Health Organization (WHO) issued a position statement that declares the development of a safe and effective group A streptococcal vaccine as one of its priorities in the fight against ARF and RHD (30). We suggest that evidence of vaccine-induced protection in the NHP model will promote the development and testing of vaccine candidates in human clinical trials. The NHP model can be easily exploited to test different vaccines, adjuvants, routes of immunization, and GAS challenge strains, and the GAS research community could greatly benefit from its utilization.

## MATERIALS AND METHODS

### Bacterial strains and growth conditions.

For recombinant protein expression, Escherichia coli BL21 Star(DE3) was grown in Luria-Bertani (LB) medium supplemented with ampicillin (100 μg ml^−1^) when appropriate. Streptococcus pyogenes M1T1 strain 5448 is an invasive clinical isolate (31), M3 strain 89437 is a pharyngitis clinical isolate (32), M12 strain HKU16 is a scarlet fever clinical isolate (33), and M28 PS001 is a puerperal sepsis clinical isolate (34). M1 strain M1:43 was used for OPK (kindly provided by S. Sriskandan). All GAS strains were routinely grown on blood agar plates and in Todd-Hewitt broth supplemented with 1% (wt/vol) yeast extract (THY). For infection experiments, a master frozen stock of GAS strain 5448 was prepared and stored at −80°C until used for infection. Briefly, GAS M1T1 strain 5448 was cultured overnight on blood agar plates at 37°C and then used to inoculate 15 ml of THY and grown overnight statically at 37°C. This starter culture was used to inoculate 100 ml of THY to an initial optical density at 600 nm (OD_600_) of 0.05 and incubated statically at 37°C until the OD_600_ reached 0.6. Bacterial cells were washed twice using ice-cold THY, resuspended in THY supplemented with 15% (vol/vol) glycerol, aliquoted, snap-frozen in liquid nitrogen, and stored at −80°C. Aliquots were thawed and used for infection and CFU enumeration.

### Antigen expression and purification.

ADI (D277A), SCPA (D130A S617A), SpyCEP (D151A S617A), and TF cloned into vector pET151/d-TOPO; SLO (P427L W535A) cloned into vector pET-15b; and M1 cloned into vector pGEX-2T were expressed in E. coli BL21 Star(DE3) cells and purified by affinity chromatography as previously described (18). Bacterial endotoxins were removed from all antigen preparations using immobilized metal ion affinity chromatography (IMAC) by supplementing washing buffers with 0.1% (vol/vol) Triton X-114 (35). All antigen preparations were filter sterilized before final formulation. For enzyme-linked immunosorbent assay (ELISA) antigens, tobacco etch virus (TEV) protease was used to cleave the His tag from ADI, TF, SCPA, and SpyCEP antigens. Uncleaved protein and TEV protease were removed using IMAC. Thrombin protease was used to cleave the His tag from SLO, and cleaved SLO was further purified using size exclusion chromatography.

### Animals.

Seventeen healthy Indian rhesus macaques (13 females and 4 males; range, 4 to 8 kg) were used in this study. All animals were housed at the Yerkes National Primate Research Center and were maintained in accordance with National Institutes of Health guidelines (36). All animal experiments were approved by the Emory University Institutional Animal Care and Use Committee.

### Immunization protocol.

NHPs were screened (day −7) to confirm the absence of a current or recent GAS infection. Throat swabs were collected and plated undiluted in Strep Selective II agar (Remel). Serum samples were collected to confirm the presence of low anti-SLO serum titers using an ASO latex test (ASI) and of anti-DNase B serum titers using an anti-DNase B antibody ELISA kit (American Research Products, Inc.) according to the manufacturer’s protocol. The immunization regime consisted of three intramuscular (i.m.) injections performed at week 0 (prime), week 8 (boost), and week 17 (boost). Two separate immunization experiments are described in this work. In the first experiment, 5 NHPs were immunized with 100 μg of M1 protein formulated in alum (Alhydrogel 2%; Brenntag Biosector), and 2 NHPs were immunized with PBS in alum, as a negative control. Serum samples were collected before each immunization to monitor antigen-specific antibody responses. Anesthesia using ketamine (10 mg/kg of body weight i.m.) or tiletamine-zolazepam (Telazol) (5 mg/kg i.m.) was used for all blood and sample collections. In the second experiment, 5 NHPs per group were immunized via intramuscular injection with 100 μg total protein of Combo5 (20 μg each antigen) or PBS as a negative control, both formulated in alum.

### Intranasal GAS infection.

Three weeks following the last booster immunization (week 20), NHPs were challenged with GAS M1T1 strain 5548. On the day of infection, individual aliquots of the GAS frozen stock were used to infect anesthetized macaques by slowly inoculating 1 ml of the GAS suspension through the nares. The infecting dose was confirmed by plating diluted aliquots of the bacterial stock onto blood agar plates. NHPs were monitored on days 1, 2, 3, 7, 14, 21, and 28 postinfection for changes in body temperature, weight gain, and white blood cell (WBC) counts. Throat swabs were collected at each time point to monitor and score GAS colonization (see Table S3 in the supplemental material). Veterinary inspection of the oropharynx was undertaken at each time point to determine tonsillitis and pharyngitis scores using an established grading system (Table S1) (14). Serum samples were collected at each time point during the course of infection. At the completion of the study (day 28 postinfection), NHPs were treated daily with penicillin G (30,000 U/kg) via intramuscular injection for 7 days. At the end of the treatment regime, throat swabs were taken to confirm the culture-negative status for GAS.

### Antigen-specific ELISA.

Antibody titers against individual antigens were determined by an ELISA. Briefly, proteins (5 μg ml^−1^) in carbonate coating buffer (50 mM Na_2_CO_3_-NaHCO_3_, pH 9.6) were adsorbed to Titertek polyvinyl chloride (PVC) microplates (MP Biomedicals) using 100 μl per well (overnight at 4°C). Plates were blocked using 5% (wt/vol) skim milk in phosphate-buffered saline containing 0.05% (vol/vol) Tween 20 (PBST) (90 min at 37°C). Plates were washed three times using PBST before the addition of NHP serum serially diluted in 0.5% (wt/vol) skim milk in PBST (90 min at 37°C). Antigen-specific NHP antibodies were detected with horseradish peroxidase (HRP)-conjugated mouse anti-monkey IgG antibody (SouthernBiotech) at a 1:4,000 dilution (90 min at 37°C). SigmaFast *o*-phenylenediamine dihydrochloride (OPD) (Sigma-Aldrich) was used as an HRP substrate, and the absorbance was measured at 450 nm. Endpoint titers were determined as the highest dilution of serum for which the absorbance was 3 standard deviations (SD) above the mean optical density of blank wells.

### Measurement of inflammation markers in NHP serum.

Inflammation markers present in sera during the course of infection were detected using the LEGENDplex nonhuman primate 13-plex inflammation panel (BioLegend) according to the manufacturer’s protocol. Samples were acquired using a BD Accuri C6 flow cytometer (BD Biosciences), and data were analyzed using LEGENDplex data analysis software (BioLegend). Data for IL-6, IP-10, IL-1β, IL-17A, IFN-β, IL-23, TNF-α, IFN-γ, GM-CSF, IL-8, and MCP-1 are presented.

### Antibody binding to the GAS surface.

GAS strains (M1, M3, M12, and M28 serotypes) were grown to mid-logarithmic phase (OD_600_ of 0.6), washed in PBS, resuspended in nonspecific human IgG (200 μg ml^−1^; Merck Millipore) in PBS with 3% (wt/vol) bovine serum albumin (BSA) (3% BSA–PBS), and incubated for 1 h at 4°C for blocking. Bacterial cells were washed and resuspended in PBS and adjusted to an OD_600_ of 0.6. Samples of 100 μl were incubated (overnight at 4°C) in triplicate with pooled NHP sera diluted 1:50 in 0.3% (wt/vol) BSA–PBS. Pellets were washed with PBS and resuspended in 100 μl of a 1:200 dilution of mouse anti-monkey IgG conjugated to Alexa Fluor 488 (SouthernBiotech) in 0.3% (wt/vol) BSA–PBS. Cells were washed with PBS and fixed in 1.5% (wt/vol) paraformaldehyde–PBS. A total of 10,000 events were acquired using a BD Accuri C6 flow cytometer (BD Biosciences).

### Opsonophagocytic killing assay.

The OPK assay was adapted for GAS (11) from that previously developed for Streptococcus pneumoniae (37). Briefly, 10 μl of GAS M1 strain M1:43, washed and diluted to 120,000 CFU per ml in opsonization buffer (10% [vol/vol] defined fetal bovine serum [FBS] [HyClone] and 0.1% [wt/vol] gelatin [Sigma-Aldrich Company Ltd.] in Hanks’ balanced salt solution [HBSS] with Ca/Mg), was incubated for 30 min at room temperature with shaking at 700 rpm on a mini-orbital shaker with 20 μl sera and serially diluted in opsonization buffer in a round-bottomed 96-well plate. Ten microliters of baby rabbit complement, diluted one in six in opsonization buffer, and 40 μl differentiated human promyelocytic leukemia cells (HL-60) were added to each dilution of serum and incubated for 90 min at 37°C with 5% CO_2_, with shaking at 700 rpm. Prior to the assay, HL-60 cells were differentiated by incubation in 0.8% dimethylformamide (DMF) at 37°C with 5% CO_2_ for 5 or 6 days and diluted in opsonization buffer to 1 × 10^7^ cells per ml. Plates were then incubated on ice for 30 min, and 10 μl from each well was spotted onto THY agar plates (Todd-Hewitt broth supplemented with 0.5% [wt/vol] yeast extract [Sigma-Aldrich Company Ltd.] and 1.5% [wt/vol] bacteriological agar [Sigma-Aldrich Company Ltd.]). An overlay agar, consisting of Todd-Hewitt broth supplemented with yeast extract, 0.75% (wt/vol) bacteriological agar, and 0.0025% 2,3,5-tetraphenyltetrazolium chloride (Sigma-Aldrich Company Ltd.), was poured onto each plate. Plates were incubated overnight at 37°C with 5% CO_2_. The number of surviving CFU was then counted using a ProtoCOL3 automated colony counter (Synbiosis, Cambridge, UK), and the dilution of sera resulting in 50% killing was calculated as the opsonic index (OI).

### Red blood cell hemolysis.

Red blood cells (RBCs) were isolated from whole human blood donated by volunteers. RBCs were separated from plasma, washed with PBS, and resuspended in the original blood volume using PBS (100% RBC solution). Combo5-alum and PBS-alum immune sera (100 μl) were serially diluted in PBS before the addition of recombinant SLO (40 ng) in the presence of 2 mM dithiothreitol (DTT). The mixture was incubated for 1 h at 37°C before the addition of 100 μl of the 4% RBC solution. Reaction mixtures were incubated for 30 min at 37°C. Samples were then centrifuged at 1,000 × *g* for 10 min, and 25 μl of the supernatant was diluted in 75 μl of PBS. The absorbance was read at 405 nm. SLO in the absence of immune sera was used as a positive control to calculate 100% hemolytic activity.

### IL-8 degradation.

Degradation of IL-8 by GAS was undertaken as previously described (38), with minor modifications. Briefly, GAS was grown to mid-logarithmic phase, washed with PBS, and resuspended in Dulbecco’s modified Eagle’s medium (DMEM) supplemented with 10% FBS to an OD_600_ of 0.4. Combo5-alum and PBS-alum immune sera were added to a final dilution of 1:100, and samples where incubated for 1 h at 4°C before the addition of 80 pg of recombinant IL-8 (BD Biosciences Pharmingen). Samples were then incubated for 2 and 4 h at 37°C. Supernatants were isolated by centrifugation and used to determine the residual IL-8 concentrations by an ELISA according to the manufacturer’s protocol (BD OptEIA).

### Statistical analyses.

Cumulative scores for colonization, pharyngitis, and tonsillitis were analyzed using the Mantel-Cox log rank test, with a *P* value of *<*0.05 considered statistically significant (GraphPad Prism 7). Log values of antigen-specific endpoint titers were analyzed using the two-tailed unpaired *t* test, with a *P* value of *<*0.05 considered statistically significant (GraphPad Prism 7). Flow cytometry data were analyzed using the probability binning algorithm in FlowJo 10.4.1 (Tree Star, Inc.), a cutoff *T*(*X*) value of 200 was experimentally determined, and samples having a *T*(*X*) value of >200 were considered significant (*P < *0.01) (99% confidence). Opsonic index values for each group were analyzed using an unpaired *t* test (GraphPad Prism 7), with a *P* value of *<*0.05 considered statistically significant (95% confidence). Residual IL-8 concentrations were compared using two-way analysis of variance (ANOVA) with Sidak’s multiple-comparison test, with a *P* value of <0.05 considered statistically significant (95% confidence).

### Ethics approvals.

All animals were maintained in accordance with National Institutes of Health guidelines (36). All animal procedures were approved by the Emory University Institutional Animal Care and Use Committee. Human blood donation for use in hemolysis assays was conducted in accordance with the *National Statement on Ethical Conduct in Human Research* (39), complied with the regulations governing experimentation on humans, and was approved by the University of Queensland Medical Research Ethics Committee.
