# Erratum for Rivera-Hernandez et al., “An Experimental Group A *Streptococcus* Vaccine That Reduces Pharyngitis and Tonsillitis in a Nonhuman Primate Model”

**DOI:** 10.1128/mBio.02995-20

**Published:** 2020-12-01

**Authors:** Tania Rivera-Hernandez, Diane G. Carnathan, Scott Jones, Amanda J. Cork, Mark R. Davies, Peter M. Moyle, Istvan Toth, Michael R. Batzloff, James McCarthy, Victor Nizet, David Goldblatt, Guido Silvestri, Mark J. Walker

**Affiliations:** a Australian Infectious Diseases Research Centre, The University of Queensland, St. Lucia, QLD, Australia; b School of Chemistry and Molecular Biosciences, The University of Queensland, St. Lucia, QLD, Australia; c Emory Vaccine Center, Emory University, Atlanta, Georgia, USA; d Yerkes National Primate Research Center, Emory University, Atlanta, Georgia, USA; e Great Ormond Street Institute of Child Health, University College London, London, United Kingdom; f Peter Doherty Institute, University of Melbourne, Parkville, VIC, Australia; g School of Pharmacy, The University of Queensland, St. Lucia, QLD, Australia; h Institute for Glycomics, Griffith University, Gold Coast, QLD, Australia; i Australian Infectious Diseases Research Centre, QIMR Berghofer Medical Research Institute, Brisbane, QLD, Australia; j Division of Host-Microbe Systems and Therapeutics, Department of Pediatrics, University of California—San Diego, La Jolla, California, USA

## ERRATUM

Volume 10, issue 2, e00693-19, 2019, https://doi.org/10.1128/mBio.00693-19. On PDF page 7, the point mutations for SCPA were incorrectly written as “SCPA (D130A S617A)” and should be “SCPA (D130A S512A)”.

