# Supplementary material for: An Experimental Group A Streptococcus Vaccine That Reduces Pharyngitis and Tonsillitis in a Nonhuman Primate Model
Source: mBio. 2019 Apr 30;10(2):e00693-19. doi: 10.1128/mBio.00693-19 (PMC6495378; doi:10.1128/mBio.00693-19)
Supplement: TABLE S1 [file mBio.00693-19-st001.pdf]

Supplementary Table 1. Pharyngitis and tonsillitis symptoms scoring system (Skinner et al. Microb Pathog 50:39-47 doi: 10.1016/j.micpath.2010.10.004).

**Pharyngitis**	**Tonsillitis**	**Score**
Normal	Normal tonsillar space, & oropharyngeal space	**0**
Mild erythema with hyperemic blood vessels	Approximate 25% increase in tonsil size; 0-25% occlusion of oropharyngeal space	**1**
More intense erythema and palatal petechiae	Approximate 50% increase in tonsil size; 25-50% occlusion of oropharyngeal space	**2**
Intense erythema with palatal petechiae and exudative tonsillitis	Approximate 75% increase in tonsil size; 50-75% occlusion of oropharyngeal space	**3**
