# Supplementary material for: An Experimental Group A Streptococcus Vaccine That Reduces Pharyngitis and Tonsillitis in a Nonhuman Primate Model
Source: mBio. 2019 Apr 30;10(2):e00693-19. doi: 10.1128/mBio.00693-19 (PMC6495378; doi:10.1128/mBio.00693-19)
Supplement: TABLE S2 [file mBio.00693-19-st002.pdf]

Supplementary Table 2. Colonization, pharyngitis and tonsillitis symptoms in pilot experiments.

	NHP ID	Symptom	Day post-infection							
			0	1	2	3	7	14	21	28
**Pilot 1** (1x10^7 CFU)	RKk15	Colonization**	-	+	+	+	+	+	+	+
		Pharyngitis	-	0	0	1	0	1	0	2
		Tonsillitis	-	0	0	0	1	1	1	2
	RUj15	Colonization**	-	-	-	-	-	-	-	-
		Pharyngitis	-	0	0	0	0	0	1	0
		Tonsillitis	-	0	0	0	0	0	2	1
**Pilot 2** (5x10^7 CFU)	RQi15	Colonization**	-	+	+	+	+	+	-	-
		Pharyngitis	0	0	1	NR*	1	1	1	0
		Tonsillitis	0	0	0	NR*	0	1	1	0
	RIp15	Colonization**	-	+	+	+	+	+	+	+
		Pharyngitis	0	0	1	1	1	1	2	0
		Tonsillitis	0	0	0	0	1	1	1	1

*NR - not recorded; ** "+" = β hemolytic colonies detected, "-" = absence
