# Supplementary material for: An Experimental Group A Streptococcus Vaccine That Reduces Pharyngitis and Tonsillitis in a Nonhuman Primate Model
Source: mBio. 2019 Apr 30;10(2):e00693-19. doi: 10.1128/mBio.00693-19 (PMC6495378; doi:10.1128/mBio.00693-19)
Supplement: FIG S1 [file mBio.00693-19-sf001.pdf]

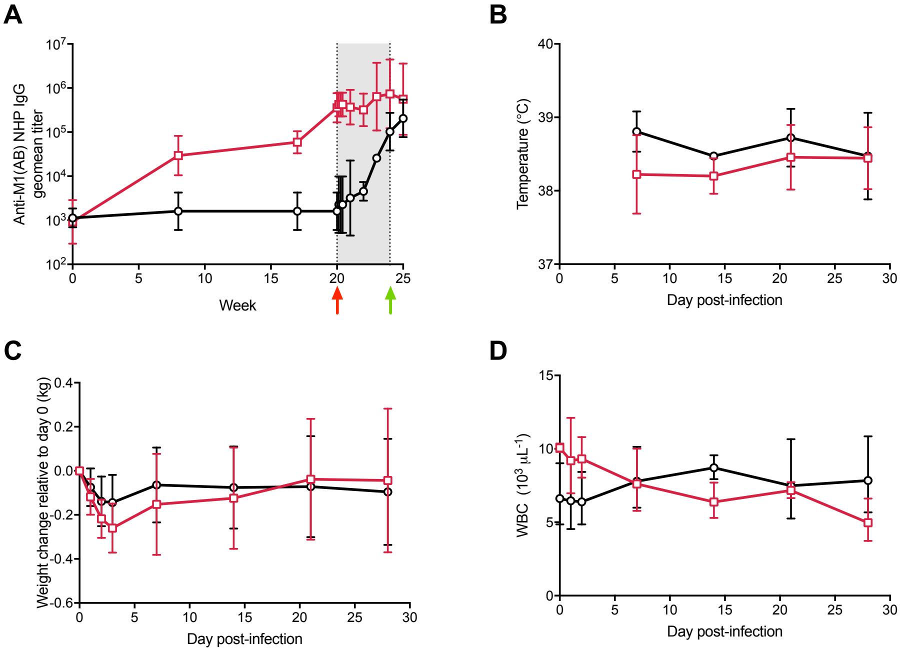

Fig. S1. Antibody response and animal welfare measures following infection of M1 immunized NHPs. (**A**) Serum samples from M1 (n=5, red line) and PBS (n=2, black line) immunized NHPs were collected before each immunization on weeks 0, 8 and 17 and post-infection on weeks 20 (days 0, 1, 2 and 3 post-infection), 21 (day 7 post-infection), 22 (day 14 post-infection), 23 (day 21 post-infection), 24 (day 28 post-infection) and 25 (one week after antibiotic treatment). Antibody titers at the time of infection (week 20) were significantly higher in M1 immunized NHPs compared to PBS immunized NHPs ($P<0.0001$). Infection and antibiotic treatment days are marked by red and green arrows respectively. Duration of GAS infection is indicated by the gray shaded area. Values represent the geometric mean titer ± geometric SD. Welfare of NHPs during the course of infection was monitored by measuring (**B**) rectal temperature, (**C**) weight, and (**D**) white blood cell counts. Symbols represent the mean values ± SD.
