# Supplementary material for: An Experimental Group A Streptococcus Vaccine That Reduces Pharyngitis and Tonsillitis in a Nonhuman Primate Model
Source: mBio. 2019 Apr 30;10(2):e00693-19. doi: 10.1128/mBio.00693-19 (PMC6495378; doi:10.1128/mBio.00693-19)
Supplement: TABLE S3 [file mBio.00693-19-st003.pdf]

Supplementary Table 3. Colonization scoring system (Dunne EM, et al. BMC Infect Dis 13:312, doi: 10.1186/1471-2334-13-312)

**Colonization**	**Score**
No β-hemolytic colonies	**0**
<10 β-hemolytic colonies in 1st quadrant	**1**
>10 β-hemolytic colonies in 1st quadrant	**2**
>10 β-hemolytic colonies in 1st and 2nd quadrants	**3**
>10 β-hemolytic colonies in 1st, 2nd and 3rd quadrants	**4**
>10 β-hemolytic colonies in 1st, 2nd, 3rd and 4th quadrants	**5**
