# Supplementary material for: An Experimental Group A Streptococcus Vaccine That Reduces Pharyngitis and Tonsillitis in a Nonhuman Primate Model
Source: mBio. 2019 Apr 30;10(2):e00693-19. doi: 10.1128/mBio.00693-19 (PMC6495378; doi:10.1128/mBio.00693-19)
Supplement: FIG S2 [file mBio.00693-19-sf002.pdf]

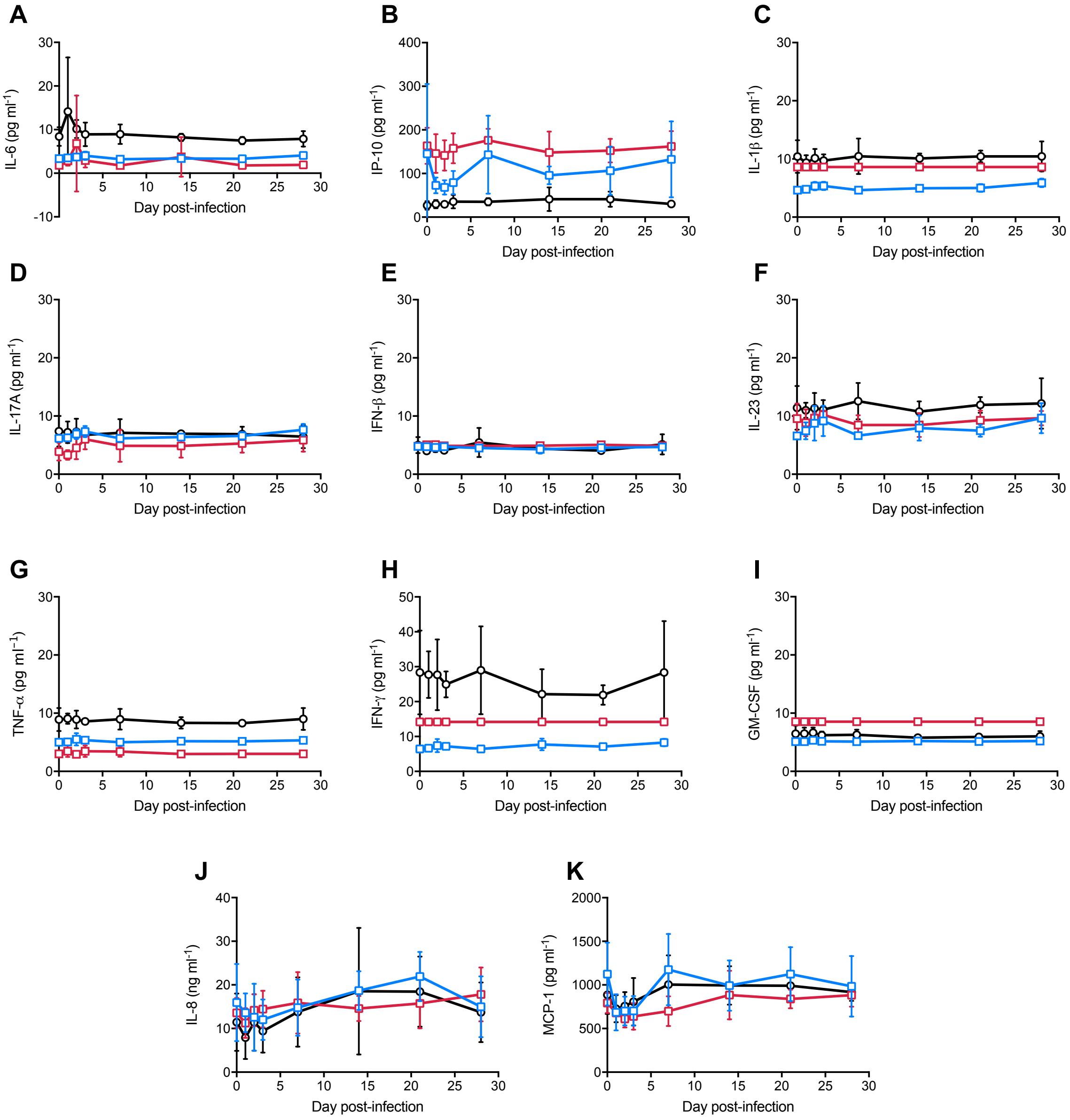

Fig. S2. Inflammation markers during GAS infection. Serum samples from Combo5 (blue line), M1 (red line) and PBS (black line) immunized NHPs collected during GAS infection were used to detect inflammation markers. A multiplex assay was used to detect (**A**) IL-6, (**B**) IP-10, (**C**) IL-1β, (**D**) IL-17A, (**E**) IFN-β, (**F**) IL-23, (**G**) TNF-α, (**H**) IFN-γ, (**I**)GM-CSF, (**J**) IL-8 and (**K**) MCP-1. Samples were analyzed by duplicate, symbols represent the mean values ± SD.
