# Supplementary material for: An Experimental Group A Streptococcus Vaccine That Reduces Pharyngitis and Tonsillitis in a Nonhuman Primate Model
Source: mBio. 2019 Apr 30;10(2):e00693-19. doi: 10.1128/mBio.00693-19 (PMC6495378; doi:10.1128/mBio.00693-19)
Supplement: FIG S3 [file mBio.00693-19-sf003.pdf]

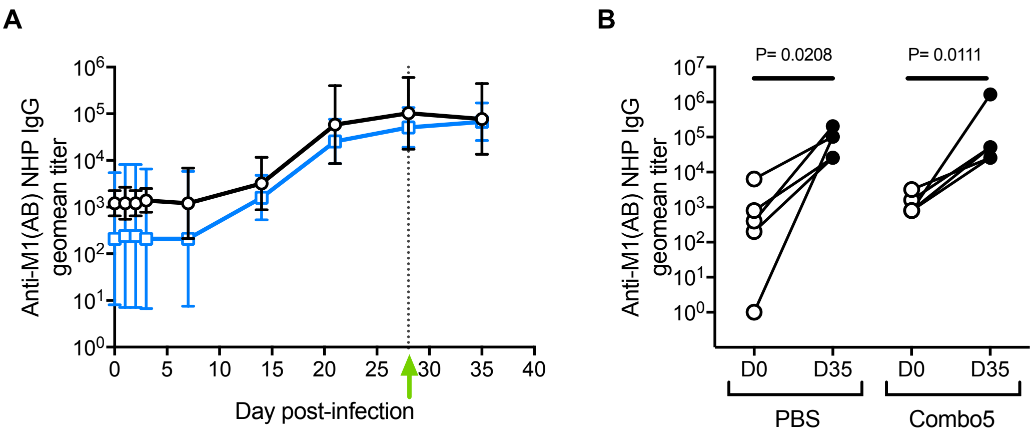

Fig. S3. Development of anti-M1 antibodies in response to infection. (**A**) Serum samples from PBS (n=5, black line) and Combo5 (n=5, blue line) immunized NHPs were collected at days 0, 1, 2, 3, 7, 14, 21, 28 and 35 post-infection. The green arrow indicates antibiotic treatment. Values represent the geometric mean titer ± geometric SD. **(B)** Anti-M1 antibody titers at the end of the experiment (day 35 post-infection) were significantly higher than prior to infection (day 0) for all NHPs in PBS and Combo5 immunized NHPs.
