# Supplementary material for: An Experimental Group A Streptococcus Vaccine That Reduces Pharyngitis and Tonsillitis in a Nonhuman Primate Model
Source: mBio. 2019 Apr 30;10(2):e00693-19. doi: 10.1128/mBio.00693-19 (PMC6495378; doi:10.1128/mBio.00693-19)
Supplement: FIG S4 [file mBio.00693-19-sf004.pdf]

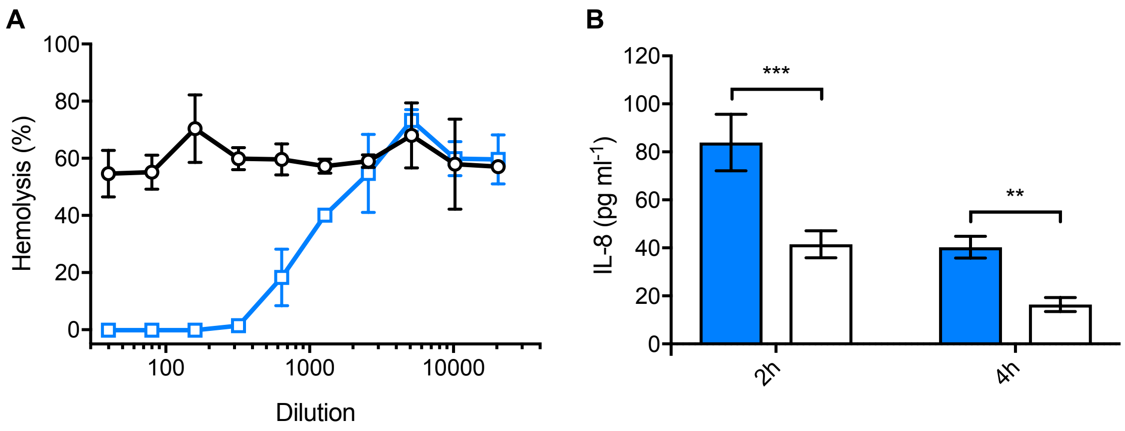

Fig. S4. Effector functions of Combo5 antibodies. (A) Pooled serum from Combo5 (blue line) immunized NHPs was able to inhibit SLO hemolytic activity compared to serum from PBS (black line) immunized NHPs. Hemolytic activity of SLO in the absence of serum was used as 100%. Values represent the mean ± SD. **(B)** Serum from Combo5 immunized NHPs (blue bars) significantly inhibited SpyCEP mediated IL-8 degradation compared to serum from PBS immunized NHPs (white bars) after 2 and 4 h co-incubation with live GAS. Bars represent the mean ± SD. Residual IL-8 concentrations were compared using a two-way ANOVA with Sidak's multiple comparisons test (**P<0.005, ***P<0.001).
